# Association between HbA1c and deep sternal wound infection after coronary artery bypass: a systematic review and meta-analysis

**DOI:** 10.1186/s13019-024-02549-6

**Published:** 2024-02-04

**Authors:** Wenyu Zhao, Jingui Xie, Zhichao Zheng, Han Zhou, Oon Cheong Ooi, Haidong Luo

**Affiliations:** 1 School of Management, University of Science and Technology of China, Hefei, China; 2 School of Management, Technical University of Munich, Heilbronn, Germany; 3 Munich Data Science Institute, Technical University of Munich, Munich, Germany; 4 Lee Kong Chian School of Business, Singapore Management University, Singapore, Singapore; 5 Department of Cardiac, Thoracic & Vascular Surgery, National University Hospital, Singapore, Singapore

**Keywords:** HbA1c, Deep sternal wound infection, DSWI, CABG, Dose-response, Meta-analysis

## Abstract

**Background:**

Deep sternal wound infection (DSWI) constitutes a serious complication after coronary artery bypass grafting (CABG) surgery. The aim of this study is to evaluate the dose-response relationship between glycated hemoglobin (HbA1c) level and the risk of DSWI after CABG.

**Methods:**

PubMed, Scopus, and Cochrane Library databases were searched to identify potentially relevant articles. According to rigorous inclusion and exclusion criteria, fourteen studies including 15,570 patients were enrolled in our meta-analysis. Odds ratio (OR) with 95% confidence intervals (CIs) was used as the summary statistic. The robust-error meta-regression model was used to synthesize the dose-response relationship.

**Results:**

Our meta-analysis shows that among patients undergoing CABG, preoperative elevated HbA1c was associated with the risk of developing DSWI (OR = 2.67, 95% CI 2.00–3.58) but with low prognostic accuracy (diagnostic OR = 2.70, 95% CI 1.96–3.73; area under the curve = 0.66, 95% CI 0.62–0.70) for predicting postoperative DSWI. Subgroup analyses showed the relationship became nonsignificant in patients without diabetes and studies adopting lower HbA1c thresholds. Dose-response analysis showed a significant nonlinear (*p* = 0.03) relationship between HbA1c and DSWI, with a significantly increased risk of DSWI when HbA1c was > 5.7%.

**Conclusions:**

An elevated HbA1c level of > 5.7% was related to a higher risk of developing DSWI after CABG, and the risk increased as the HbA1c level grew. The association between HbA1c and DSWI was nonsignificant among nondiabetic patients while significant among diabetic patients.

## Background

Deep sternal wound infection (DSWI) is a severe complication after CABG surgery, which has potentially devastating consequences. DSWI may lead to life-threatening complications with an increase in mortality, morbidity, and healthcare cost [1–3]. Therefore, it is necessary to identify the risk factors for developing DSWI.

After CABG, diabetes mellitus is associated with an increased risk of infection [4], and glycemic control plays a vital role in the immune response of patients with diabetes [5]. Abnormal blood glucose level might be an essential predictor of the development of DSWI in patients undergoing CABG. However, fasting glucose is prone to be unstable due to various factors [6], the long-term state of blood glucose level could better reflect glycemic control status. HbA1c is used to assess long-term glycemic control, which indicates the average long-term glucose metabolic state during a period of 2 to 3 months before surgery [7]. Practically, it is important to assess the effectiveness of elevated HbA1c levels preoperatively for the prediction of DSWI development after CABG, especially in patients diagnosed with diabetes.

Several studies have explored the relationship between elevated HbA1c levels and the risk of DSWI. The 2011 ACCF/AHA Guideline for CABG recommends the use of continuous intravenous insulin to achieve and maintain an early postoperative blood glucose concentration of ≤ 180 mg/dL as a measure to reduce the incidence of DSWI [8]. One study involving 3,555 patients undergoing CABG treated HbA1c levels as a continuous variable and, through multivariable logistic regression, found a significant association between elevated HbA1c levels and an increased risk of DSWI [9]. Another retrospective study used multivariate analysis to indicate that an HbA1c level of 6.5% or higher in patients undergoing CABG was associated with a significant increase in the risk of DSWI [10]. However, given the variations in patient demographics, HbA1c levels, and study design, a comprehensive systematic review on the topic is warranted.

The primary objective of this study was to evaluate the predictive value of elevated preoperative HbA1c levels for developing DSWI (including mediastinitis) in patients undergoing CABG. We also performed analysis across different subgroups of patients. In addition, we conducted a dose-response meta-analysis to investigate the association between HbA1c levels and DSWI.

## Methods

### Search strategy and study selection

We searched published studies in PubMed, Scopus, and Cochrane Library from inception to February 2023, using the following search terms: (“HgbA1C” OR “HbA1c” OR “glycosylated hemoglobin” OR “glycated hemoglobin” OR “hemoglobin A1c”) AND (“Coronary Bypass Graft” OR “coronary artery bypass grafting” OR “coronary artery bypass surgery” OR “CABG”). DSWI-related terms were not used in our search in case missing studies that assessed the relationship between elevated HbA1c and many adverse outcomes, not just DSWI.

The inclusion criteria were as follows: (1) observational cohort or case-control studies, (2) studies reported the association between preoperative HbA1c levels and DSWI, and (3) studies with sufficient data to evaluate OR with 95% confidence intervals. For studies with overlapping data, our meta-analysis only included the study with more complete surgical data.

### Data extraction and quality assessment

Using a standardized data collection form, we extracted the following data from each study: first author, publication year, country, study design, length of follow-up, sample size, baseline characteristics, and HbA1c threshold. We adopted the Newcastle–Ottawa scale (NOS)—a commonly used tool to assess case-control studies and cohort studies with a total score of 9 stars [11]—to evaluate the quality of each study. We considered studies with a score greater than 6 to be of high quality.

### Statistical analysis

In our pooled analysis, we aimed to investigate the association between elevated HbA1c and DSWI in patients undergoing CABG and evaluate the accuracy of elevated HbA1c in predicting DSWI. We followed the common practice in the literature and defined elevated HbA1c as being above a specified threshold. For a study with more than one threshold, we chose either 6.5% or 7.0% as the threshold, as they were most widely reported in the literature, details would be explained later. The ORs with 95% CIs were used to evaluate the association, they were either directly extracted or calculated from reported DSWI incidence. In addition, we conducted a diagnostic meta-analysis to estimate the diagnostic odds ratio (DOR). The area under the receiver-operating characteristic curve (AUC) was used to assess the discriminative power of elevated HbA1c for predicting DSWI.

We further performed subgroup analyses based on multiple variables, including study design, sample size, study period, patient type, and threshold level. Next, we attempted to establish a dose-response relationship between HbA1c and DSWI. Because most of the included studies reported only two-category data, the robust-error meta-regression model was used to synthesize the dose-response relationship across available studies [12]. A nonlinear curve was fitted by the restricted cubic spline in the regression [13], and nonlinearity was tested using the Wald test. The assigned values were selected in the following order: (1) the mean value in each category of HbA1c level, (2) the midpoint of the upper and lower boundaries in each category of HbA1c level, and (3) the reported boundary multiplied or divided by 1.25 [14].

We analyzed the heterogeneity among the included studies using Cochran’s Q test and the ${I}^{2}$ test. We considered heterogeneity to be significant if *p* < 0.1 for the Q statistic. An ${I}^{2}$ greater than 50% indicates substantial heterogeneity, in which case a random-effects model would be used [15]. Otherwise, we used a fixed-effects model. The source of heterogeneity was explored via sensitivity analyses. Moreover, we used Begg’s test to evaluate publication bias. A *p* value < 0.05 was considered to be statistically significant. All *p* values were two sided. All statistical analyses were performed using Stata statistical software (version 17.0).

## Results

### Search results

**Fig. 1:**
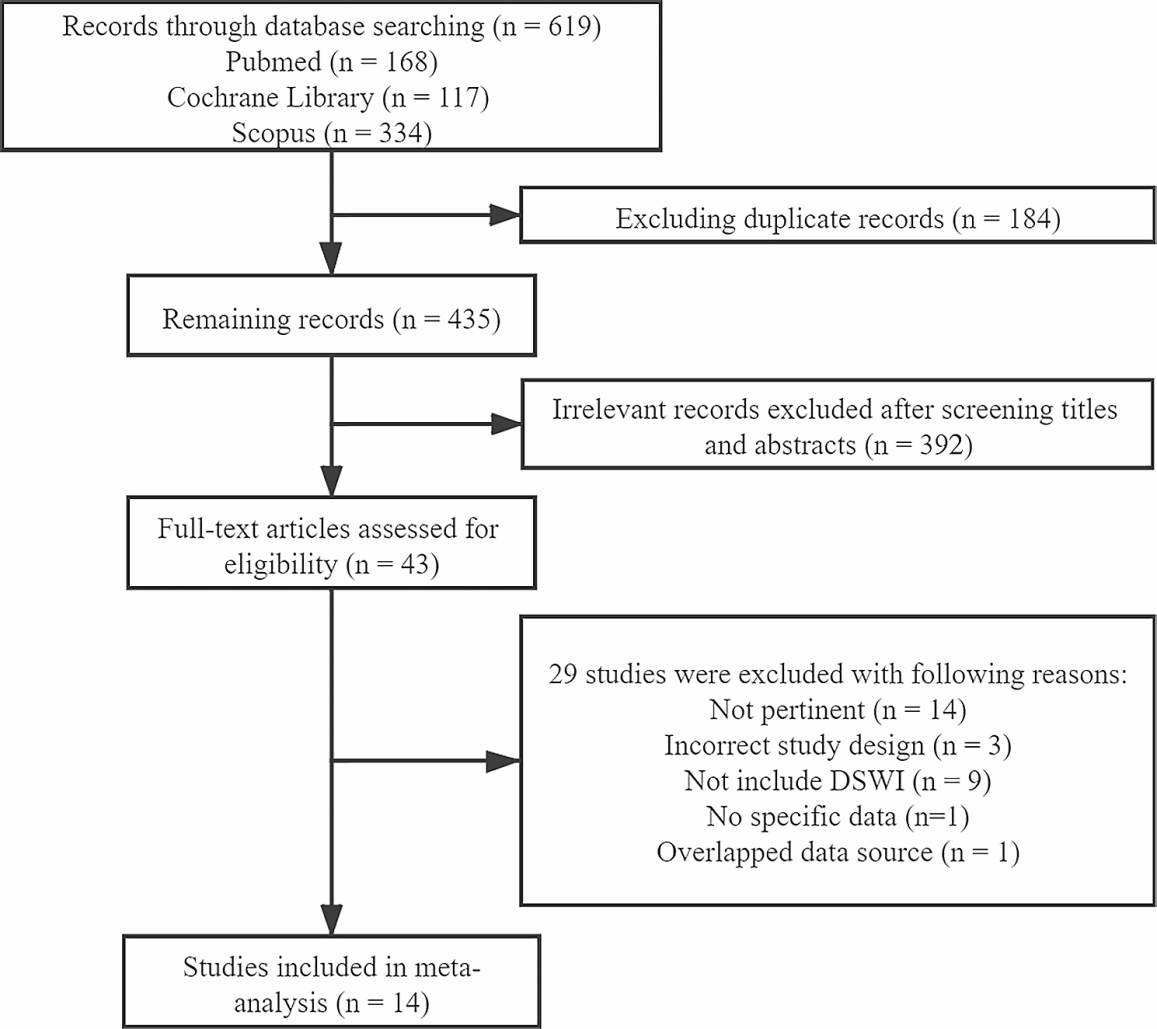
Flow diagram of study selection process

The flowchart in Fig. 1 illustrates the literature search and selection process. A total of 619 published articles were preliminarily identified (168 from PubMed, 117 from the Cochrane Library, and 334 from Scopus). After deleting duplicate reports, we screened 435 articles that potentially met the requirements. 392 of 435 were irrelevant and excluded. We further reviewed the remaining 43 potential studies, and 14 met our inclusion criteria.

### Description of included studies

Table 1 summarizes the characteristics of the 14 included studies. A total of 15,570 subjects were included in our analysis. The number of participants in each study ranged from 80 to 4,678, and the proportion of males ranged from 70.98 to 90.94%. The average age of patients ranged from 57.3 to 68 years, and their average body mass index (BMI) varied between 23.8 and 31. Among patients with normal HbA1c, the incidence of DSWI ranged from 0 to 2.73%, and in patients with elevated HbA1c, it ranged from 0 to 6.25%. The cutoff points for preoperative HbA1c levels ranged from 5.0 to 9.0%: 9 of the 14 studies used either 6.5% or 7.0% as the only threshold, and one used 5.9% as the only threshold; the other four studies used two thresholds, one of which was either 6.5% or 7.0%. For the four studies with two thresholds, we chose 6.5% or 7.0% (whichever was one of the two thresholds) as the only threshold and consolidated the results from three HbA1c categories into two categories. 5 of the 14 studies only included patients with diabetes, and the rest included both diabetic and nondiabetic patients. Of the selected studies, the NOS scores were greater than 6 in all but two of the included studies (with scores of 5). In general, the quality of the studies was considered satisfactory. To mitigate the confounding effect of urgent procedures on postoperative outcomes, and considering that preoperative HbA1c data is typically available only before elective surgeries, only three studies included emergency surgical patients [16–18]. The proportion of emergency patients in these studies ranged from 1.4 to 13.1%.

**Table 1:** Characteristics of included studies in the meta-analysis

Author, year [reference]	Country	Study period	Follow-up duration	Design	No. of patients	DM (%)	Male (%)	Age	Body mass index	Emer- gency (%)	Incidence of DSWI (normal HbA1c/elevated HbA1c, %)	HbA1c cutoff points	NOS
Alserius et al., 2008 [30]	Sweden	2002 to 2004	3.5 ± 0.9 years	P	605	27	79	66	31	0	1.08/1.75	6.0%; 7.0%	8
Halkos et al., 2008 [31]	United States	2002 to 2006	In-hospital stay	R	3,089	40.1	72.6	62.6	NA	0	2.13/1.85	7.0%	6
Matsuura et al., 2009 [20]	Japan	2000 to 2007	2.4 ± 1.6 years	R	101	100	79.2	65.5	24.2	NA	0/1.05	6.5%	7
Göksedef et al., 2010 [19]	Turkey	2007 to 2008	In-hospital stay/30 days	P	150	35.3	72.7	62.1	27.8	0	2.42/2.94	7.0%	8
Tsuruta et al., 2011 [32]	Japan	2002 to 2007	Perioperative	P	306	100	79.1	59.8	24.0	9.4	1.38/1.02	6.5%; 7.5%	7
Gumus et al., 2013 [33]	Turkey	2010 to 2012	NA	R	510	40.2	74.9	60.6	27.6	13.1	0.66/0	5.9%	7
Engoren et al., 2014 [18]	United States	2007 to 2010	In-hospital stay/30 days	R	880	47.2	72.7	64.7	NA	NA	0.50/2.18	6.0%; 7.0%	6
Subramaniam et al., 2014 [25]	Israel	2008 to 2011	In-hospital stay	P	1,461	38.5	74.8	68	NA	NA	0/5.17	6.5%	7
Santos et al., 2015 [34]	Argentina	NA	In-hospital stay	P	96	100	82.3	63	28	0	0.53/1.32	7.0%	6
Narayan et al., 2017 [10]	India	2011 to 2014	NA	R	4,678	65.1	90.9	58.9	23.8	0	1.28/3.68	6.5%	5
Nicolini et al., 2018 [9]	European	2015 to 2016	In-hospital stay	P	2,606	36.1	86	67.5	27.8	0	0/5.00	7.0%; 9.0%	8
Ramadan et al., 2018 [35]	Egypt	2013 to 2015	In-hospital stay	P	80	100	76.3	57.3	NA	0	2.73/4.62	7.0%	6
Almogati et al., 2019 [36]	Saudi Arabia	2013 to 2015	NA	R	305	81.6	82	59.1	27.8	NA	2.44/6.25	7.0%	5
Kim et al., 2020 [37]	Korea	2005 to 2017	30 days	R	703	100	71	65.8	NA	1.4	0.44/2.33	7.0%	6

Note: DM, diabetes mellitus; DSWI, deep sternal wound infection; HbA1c, hemoglobin A1c; P, prospective study; R, retrospective study; NA, not available

### Elevated HbA1c and DSWI

**Fig. 2:**
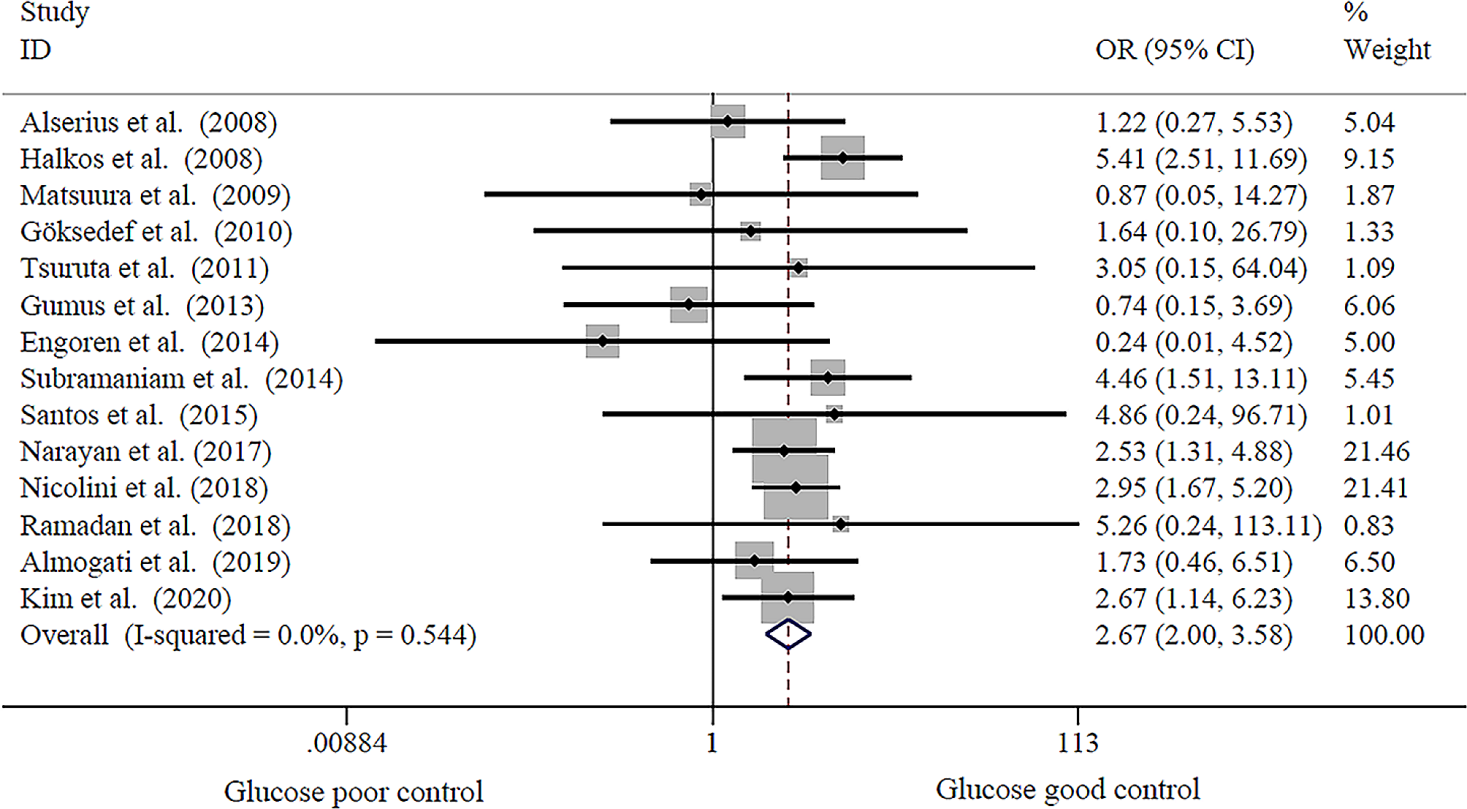
Forest plot of included studies comparing risk of postoperative DSWI between patients with poor and good glucose control (overall OR and 95% CI represented by diamond data marker)

The results shown in Fig. 2 revealed a significant correlation between preoperative HbA1c levels and the risk of developing DSWI (OR = 2.67, 95% CI 2.00–3.58, *p* = 0.00), with low heterogeneity (I^2^ = 0.00%; *p* = 0.54). In the sensitivity analysis conducted by omitting one study at a time, there was no significant change in the pooled results. No publication bias (*p* = 0.51) was detected for the primary analyses. Diagnostic test meta-analysis demonstrated that the evaluated HbA1c has a prognostic value of DSWI (DOR = 2.70, 95% CI 1.96–3.73) but with low discriminative power (AUC = 0.66, 95% CI 0.62–0.70). Therefore, elevated HbA1c was a risk factor for DSWI after CABG but had poor diagnostic performance for predicting DSWI.

### Subgroup analysis

**Fig. 3:**
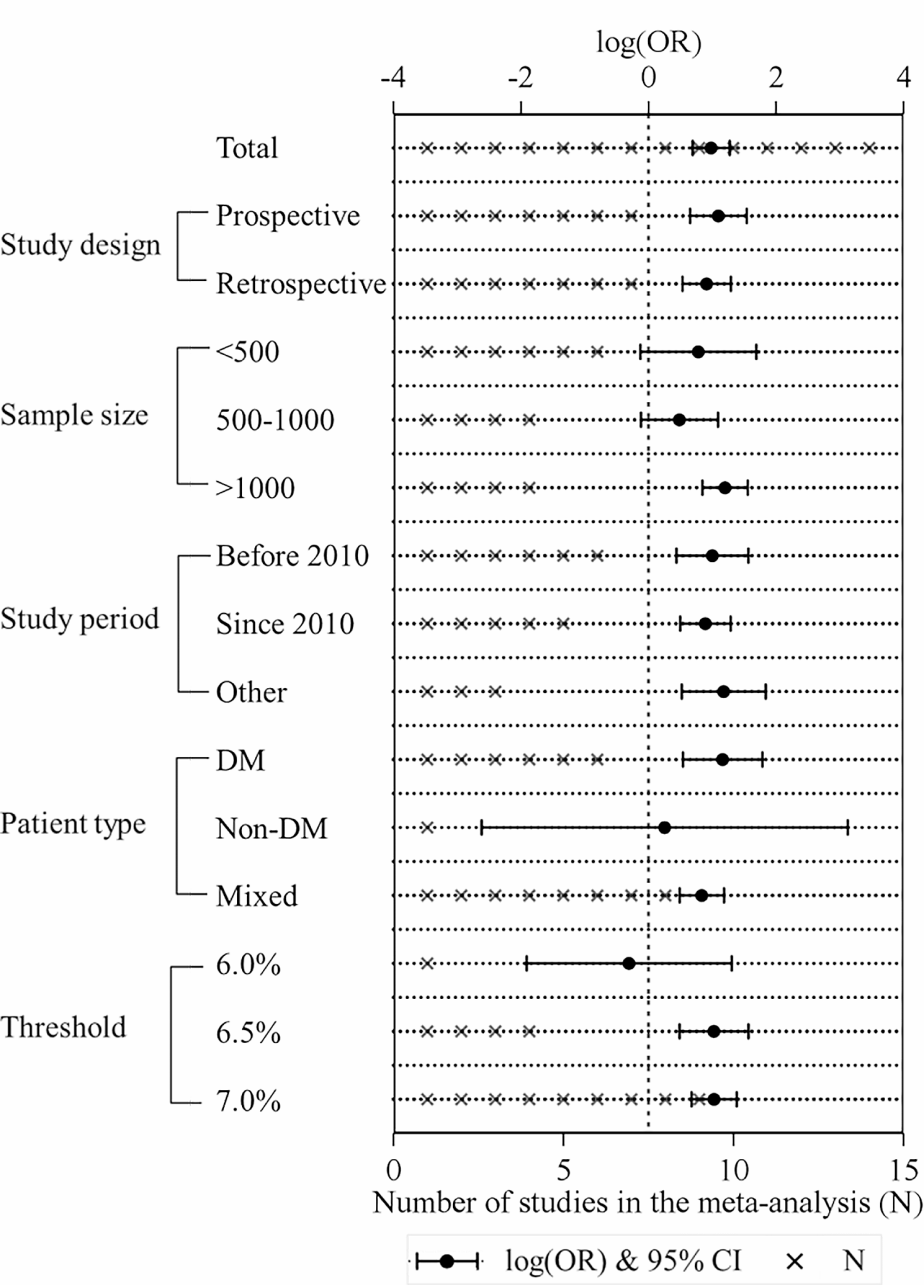
Subgroup analysis: OR and 95% CI for the outcome

Subgroup analysis (Fig. 3) indicated that the significance of the primary result was not altered by study design (prospective study: OR = 3.00, 95% CI 1.92–4.67; retrospective study: OR = 2.49, 95% CI 1.71–3.64) or study period (before 2010: OR = 2.73, 95% CI 1.55–4.79; 2010 until present: OR = 2.44, 95% CI 1.65–3.63). However, in the pooled analysis, the association became nonsignificant among patients without diabetes (patients without diabetes: OR = 1.29, 95% CI 0.07–22.78; patients with diabetes: OR = 3.20, 95% CI 1.72–5.97; mixed: OR = 2.31, 95% CI 1.63–3.27), and studies adopting a lower HbA1c threshold (6.0%: OR = 0.74, 95% CI 0.15–3.69; 6.5% OR = 2.80, 95% CI 1.63–4.80; 7.0% OR = 2.80, 95% CI 1.97–3.99). In addition, we noticed that elevated HbA1c was more strongly associated with the risk of DSWI in patients with diabetes and among studies adopting HbA1c 7.0% as the threshold.

### Dose-response association between HbA1c levels and DSWI

**Fig. 4:**
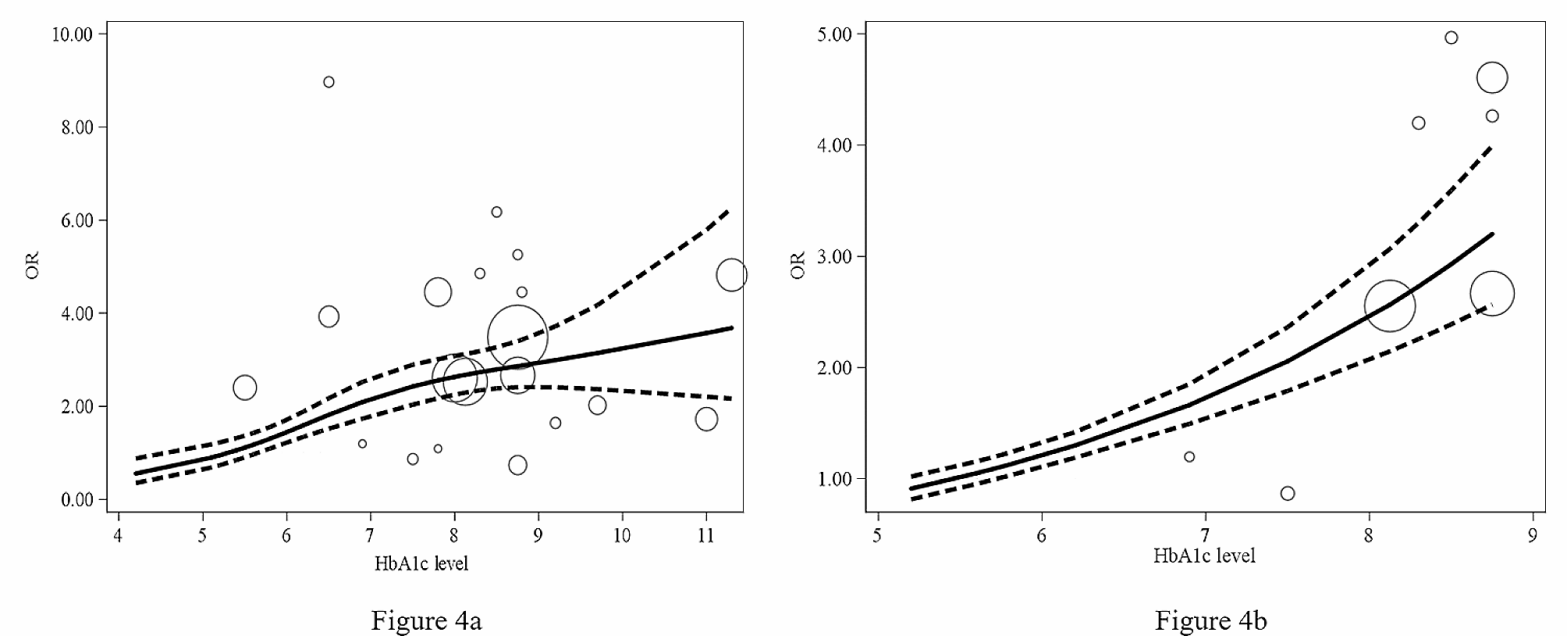
Dose-response analysis curve: relationship between HbA1c level and risk of DSWI. **4a**. All studies. **4b**. Patients diagnosed with DM

The dose-response analyses (Fig. 4a) revealed a significant nonlinear association (*p* = 0.03) between HbA1c level and risk of DSWI. The curve showed that an HbA1c greater than 5.7% significantly increased the risk of DSWI. As the HbA1c level increased, the risk of DSWI also gradually increased, and when HbA1c was between 5.7% and 7.0%, the risk of DSWI increased faster than other levels. Because the previous subgroup analysis showed no significant association between HbA1c and DSWI in patients without diabetes, we repeated the dose-response analysis in patients diagnosed with diabetes. The results revealed a linear association between HbA1c level and risks of DSWI in subjects with diabetes (Fig. 4b), and an HbA1c greater than 5.8% significantly increased the risk of DSWI. Each percentage point increase in HbA1c was associated with an increase of 1.42 in the OR of DSWI (95% CI 1.29–1.57, *p* = 0.00).

## Discussion

We conducted this systematic review and meta-analysis to assess the predictive value of the preoperative HbA1c level for developing DSWI in patients undergoing CABG, taking into consideration different confounding factors. We analyzed 14 studies on HbA1c levels and DSWI after CABG in our meta-analysis, with a total of 15,570 patients. The results demonstrated that elevated HbA1c level was associated with DSWI risk after CABG. In addition, we found that the association between HbA1c and DSWI was nonsignificant in patients without diabetes and studies that adopted lower HbA1c thresholds. In addition, the association was stronger among patients with diabetes. The dose-response analyses suggested that patients with preoperative HbA1c levels greater than 5.7% were more likely to develop DSWI.

Hyperglycemia impairs the immune system, thereby increasing the risk of infection and impeding normal wound healing [16]. The findings of related studies have been inconsistent [19–21]. Such heterogeneity could stem from multiple confounders, including patient demographics, HbA1c levels, and study design. Some studies have focused on the relationship between elevated preoperative HbA1c and diabetes, but it remains unclear whether the same relationship exists for nondiabetic patients. Additionally, many studies treated HbA1c levels as a dichotomous variable, but the thresholds for HbA1c varied across different studies [22–24]. Studies treating HbA1c as a continuous variable found that a 1% increase in preoperative HbA1c level was associated with increased complications [9, 25]. Our results demonstrated that elevated HbA1c is a risk factor for adverse outcomes after CABG. A meta-analysis [26] published in 2019 also established that patients with elevated HbA1c levels greater than 6–7% had an increased risk of developing sternal wound infection after adult cardiac surgery. Nevertheless, we unveil the insignificant relationship between HbA1c levels and DSWI risk in nondiabetic patients. Highlighting the differences between diabetic and nondiabetic patients is crucial for informing clinical decision-making. Moreover, our dose-response analysis indicated a significant nonlinear relationship between HbA1c levels and DSWI risk. The diagnostic test meta-analysis showed that a single indicator of elevated HbA1c was of little informational value in predicting DSWI (DOR = 2.70, 95% CI 1.96–3.73; AUC = 0.66, 95% CI 0.62–0.70). Given that many CABG surgeries are urgent [27], when making decisions regarding the timing of CABG based on the presumed level of glycemic control, other factors that may influence postoperative outcomes should also be taken into account.

When we performed the meta-analysis among different subgroups, we found that the association between elevated HbA1c and DSWI was not altered by study design and study period. However, the positive association in the pooled analysis became nonsignificant in small-sample studies and in studies adopting lower HbA1c thresholds. A plausible reason for this finding was that the incidence of DSWI was very low, and the incidence of DSWI in some groups was even 0. Thus, there was not enough statistical power in the small-sample studies to demonstrate a statistically significant association between elevated HbA1c and risk of DSWI [28]. Similarly, low-threshold values led to subsamples with smaller sizes, resulting in a nonsignificant association between elevation of HbA1c and DSWI.

In addition, we also found that elevated HbA1c was not a risk factor for postoperative DSWI for among patients without diabetes undergoing CABG. The association between HbA1c and DSWI was stronger in patients with diabetes than in patients with a mixture of diabetes and nondiabetes. However, we should interpret these results with caution. Elevated HbA1c was typical among patients undergoing CABG surgery, regardless of their history of diabetes [18, 29], but only a limited number of studies focused only on patients without diabetes. In our included studies, only one study provided sufficient data to perform the analysis on patients without diabetes. Although our results indicated no significant relationship between elevated HbA1c and DSWI in patients without diabetes, further studies with large sample sizes and different thresholds are expected to confirm the findings.

Furthermore, our results suggested that increased HbA1c levels were associated with an increased risk of DSWI using the dose-response method. We found a significant nonlinear association (*p* = 0.03) between HbA1c level and the risk of DSWI. The curve showed that patients with preoperative HbA1c levels greater than 5.7% were more likely to develop DSWI, and the risk increased as the HbA1c level increased. The curve also suggested that when the HbA1c level was between 5.7% and 7.0%, the risk of DSWI increased at a faster rate. This might be attributed to the treatment bias in the retrospective studies. Because a patient with an HbA1c level of 7.0% or higher was typically considered risky and thus might receive more attention or intervention, the rate of increase in the incidence of DSWI would consequently drop as a result of more active management. These results suggest that for patients with preoperative HbA1c > 5.7%, especially diabetic patients, extra care is needed to prevent DSWI after CABG.

Our study had several limitations. First, the included studies differed in country, follow-up times, internal mammary artery type, definitions of DSWI, rates of diabetes, obesity, and other comorbidities, which could result in assessment bias. Second, in the dose-response analysis, we used the assigned values of the categorical HbA1c data, which provided only an approximation of this dose-specific relationship. Third, because of the limited number of included studies, not every subgroup analysis was performed adequately.

## Conclusions

Our meta-analysis demonstrated that elevated preoperative HbA1c level was a risk factor for DSWI after CABG, but it showed poor prognostic accuracy in predicting postoperative DSWI. Subgroup analyses indicated that the association became nonsignificant in small-sample studies, patients without diabetes, and studies that adopted lower HbA1c thresholds. The association between HbA1c and DSWI was stronger in patients with diabetes. In addition, there was a significant nonlinear association between HbA1c level and DSWI risk. Patients with preoperative HbA1c levels greater than 5.7% might be more susceptible to DSWI, and the risk increased at a faster rate when HbA1c levels were between 5.7% and 7.0%.

## Data Availability

The datasets used and/or analysed during the current study are available from the corresponding author on reasonable request.

## Abbreviations

AUC: Area under the receiver-operating characteristic curve
CABG: Coronary artery bypass grafting
CI: Confidence interval
DOR: Diagnostic odds ratio
DSWI: Deep sternal wound infection
HbA1c: Hemoglobin A1c
NOS: Newcastle–Ottawa scale
OR: Odds ratio
